# Single-Operator Cholangioscopy-Guided Electrohydraulic Lithotripsy for Difficult Common Bile Duct Stones: A Prospective Cohort Study of Efficacy, Safety, and Clinical Outcomes

**DOI:** 10.14740/jocmr6650

**Published:** 2026-10-23

**Authors:** Mahmod Ghoname, Mohammad Abd Elhameed Alwaseef, Ahmed Shaaban, Sherif Mohamed Elsayed Amin Hegab, Ahmed Fawzy Selim, Mohammed Zain Abdelwadoud, Tayseer S. Karoof, Noha Fawzi Alrowaithy, Sayed Ahmed Sayed, Ashraf Ahmed Mohamed Aboismail, Yasser Rabie Abdulaziz Taalab, Housam Ahmad Helmy, Abdulmajeed Albarrak, Saad S. Alkhowaiter, Mohamed Galal Flefel

**Affiliations:** a Department of Hepatology, Gastroenterology and Infectious Diseases, Faculty of Medicine, Al-Azhar University, Cairo, Egypt; b Department of Clinical Pathology, Faculty of Medicine, Al-Azhar University, Cairo, Egypt; c Department of Anesthesia, ICU and Pain Management, Faculty of Medicine, Ain Shams University, Cairo, Egypt; d Department of Hepatology, Gastroenterology and Infectious Diseases, Ahmed Maher Teaching Hospital, Cairo, Egypt; e Department of Internal Medicine, Gastroenterology and Hepatology Unit, Faculty of Medicine, Tanta University, Tanta, Egypt; f Department of Geriatrics and Gerontology, Faculty of Medicine, Ain Shams University, Cairo, Egypt; g Department of Internal Medicine, Faculty of Medicine, Benha University, Benha, Egypt; h Department of Biochemistry, King Abdulaziz University, Jeddah, Saudi Arabia; i Laboratory Department, King Abdulaziz Medical City, Jeddah, Saudi Arabia; j Department of Tropical Medicine, Faculty of Medicine, Ain Shams University, Cairo, Egypt; k Department of Tropical Medicine, Faculty of Medicine, Alexandria University, Alexandria, Egypt; l Department of Gastroenterology, Hepatology and Infectious Diseases, Faculty of Medicine, Al-Azhar University, Damietta, Egypt; m Department of Hepatology and Gastroenterology, National Liver Institute, Menoufia University, Shebin El-Kom, Egypt; n Department of Medicine, College of Medicine, Qassim University, Saudi Arabia; o Department of Medicine, Division of Gastroenterology, College of Medicine, King Saud University, Riyadh, Saudi Arabia; p Department of Internal Medicine, Faculty of Medicine, Tanta University, Tanta, Egypt

**Keywords:** Single-operator cholangioscopy, SpyGlass, Electrohydraulic lithotripsy, Difficult common bile duct stones, ERCP, Biliary lithotripsy, Endoscopic management

## Abstract

**Background:**

Difficult common bile duct (CBD) stones, characterized by a diameter exceeding 15 mm, impaction, multiple stone burden, or associated biliary strictures, constitute 10–15% of endoscopic retrograde cholangiopancreatography (ERCP) cases and often resist conventional extraction. The application of single-operator cholangioscopy (SOC)–based electrohydraulic lithotripsy (EHL) has shown positive results as an advanced treatment method, although its real-world effectiveness needs further exploration in settings that lack resources. The researchers conducted a prospective study to assess the safety and effectiveness of SOC-based EHL treatment for patients with difficult CBD stones who did not respond to standard ERCP methods.

**Methods:**

The researchers conducted a prospective single-arm cohort study at Al-Azhar University Hospitals, which started in November 2023 and ended in July 2024 after they enrolled 25 consecutive patients who had failed standard extraction methods for difficult CBD stone removal. The patients received SOC-guided EHL treatment through the SpyGlass system, which enabled them to retrieve stone fragments using either balloon sweepers or Dormia baskets. The primary outcome tracked complete stone clearance through occlusion cholangiography, while the secondary outcomes measured three different factors, which included procedural success, assessment of ERCP sessions, and complication rates.

**Results:**

The study enrolled 25 patients who consisted of 14 females and 11 males with an average age of 61.8 years and a standard deviation of 6.4 years. The study found that the mean largest stone diameter measured 15.6 mm with a standard deviation of 3.3 mm, while 14 patients (56.0%) had multiple stones and 15 patients (60.0%) had stones that exceeded 15 mm in size. The study achieved complete ductal clearance for 22 patients (88.0%) while performing balloon extraction in 22 cases (88.0%) and using controlled radial expansion balloon dilation as an adjunct in five cases (20.0%). The study found no major complications, which included post-ERCP pancreatitis, bleeding, and perforation.

**Conclusion:**

The SOC-guided EHL system achieves high success rates through 88.0% stone elimination while maintaining a safe operational record, which enables its use as a primary treatment method for complex endoscopic procedures that decrease the need for surgical or percutaneous methods.

## Introduction

Choledocholithiasis occurs when gallstones block the common bile duct (CBD), which constitutes one of the most common and serious medical conditions that doctors diagnose in patients who require gastroenterology and hepatobiliary surgical treatment throughout the world. Doctors can treat most CBD stones successfully through standard endoscopic retrograde cholangiopancreatography (ERCP) methods, which use standard biliary sphincterotomy and balloon or basket extraction, but some patients develop “difficult” bile duct stones which do not respond to common treatment methods [1]. The difficult stones display their characteristic size when they reach diameters larger than 15 mm and they need to be removed from the bile duct because patients have multiple stone burdens and tapered distal CBD biliary strictures, and their stone-to-duct diameter ratio exceeds 1 [2]. The management of such complex stone disease presents doctors with significant technical obstacles, which lead to increased failure rates for procedures, higher rates of complications, longer patient stays in hospitals, and rising costs for healthcare services [3].

Tertiary referral centers face a major clinical challenge because difficult CBD stones occur in about 10% to 15% of ERCP procedures, which treat biliary stone disease [1]. When conventional extraction methods fail, several alternative therapeutic strategies have been traditionally employed, including mechanical lithotripsy, extracorporeal shock wave lithotripsy (ESWL), and surgical bile duct exploration. The three treatment methods show different challenges because mechanical lithotripsy fails to treat large or impacted stones, and it has a basket entrapment hazard, while ESWL needs special devices and shows success rates between 55% and 90%, and surgical exploration requires invasive procedures, which result in high morbidity and mortality for elderly patients or those with existing medical conditions [4].

Single-operator cholangioscopy (SOC) has become a fundamental development for treating complex biliary stone conditions since its introduction 10 years ago. The SpyGlass system (Boston Scientific, Marlborough, MA, USA), the most widely used SOC platform, enables direct visual inspection of the biliary tree through a dedicated cholangioscopy that is introduced through the working channel of a standard duodenoscope [5]. This direct visualization capability allows for precise targeting of lithotripsy energy onto the stone surface under direct endoscopic guidance, which is particularly advantageous when dealing with large, impacted, or intrahepatic stones that cannot be adequately visualized fluoroscopically [6]. The EHL system, combined with SOC, presents an effective treatment method which uses shock waves to break up stones into smaller pieces that doctors can see through cholangioscopy examination [2].

The research study investigates the effectiveness, safety, and operational results of SOC-guided lithotripsy through its implementation in real-world settings, yet it lacks sufficient evidence, especially from Middle Eastern and North African medical establishments. The existing knowledge base consists of evidence that comes from studies which retrospectively analyzed data and from case series that examined single medical facilities which operated in Western and East Asian regions [1, 5]. The existing body of research has not yet determined the ideal methods for choosing patients, executing procedures, and identifying factors that lead to treatment failure in SOC-guided EHL. The gap in knowledge requires clinical attention because the discovery of factors that lead to procedural failure will enhance treatment algorithms and enable better patient guidance. Current European Society of Gastrointestinal Endoscopy (ESGE) guidelines and a recent systematic review and meta-analysis report pooled complete stone clearance rates of approximately 94% with SOC-guided lithotripsy across large multicenter cohorts, underscoring its established role in the management algorithm for difficult CBD stones, although data from real-world, resource-limited settings such as ours remain comparatively scarce [7, 8].

We performed a prospective single-arm cohort study to assess both the effectiveness and safety of SOC-guided EHL treatment for challenging CBD stones. The study’s primary objective focused on measuring complete biliary duct clearance rates, which followed SOC-guided EHL treatment. The secondary objectives of the study assessed safety results, procedural details, the number of required ERCP sessions, and the use of emergency treatment methods. Our hypothesis stated that SOC-guided EHL would enable this difficult patient group to attain complete stone removal results while maintaining safe treatment standards.

## Materials and Methods

### Study design and setting

The Endoscopy Unit at Al-Azhar University Hospitals in Cairo, Egypt, conducted this single-arm cohort study from November 2023 until July 2024. The study protocol received approval from the Institutional Ethics Committee of Al-Azhar University Faculty of Medicine (IRB No. 000186), and the research followed the ethical guidelines established by the Declaration of Helsinki and its subsequent amendments [9]. All participants in the study provided written informed consent before the researchers conducted any study-related procedures. The Strengthening the Reporting of Observational Studies in Epidemiology (STROBE) guidelines for prospective cohort studies serve as the framework for this study’s reporting.

### Study population

The study included all patients who experienced difficult CBD stone problems after unsuccessfully trying standard ERCP stone extraction methods. The study required that participants with difficult CBD stones meet at least one of the following conditions: (1) stone diameter larger than 15–20 mm, (2) three or more stones within the biliary tree, (3) impacted stones refractory to standard extraction attempts, (4) stones located above biliary strictures, (5) failed conventional ERCP extraction despite adequate sphincterotomy, and (6) stone-to-CBD diameter ratio greater than 1. The study excluded participants who had uncorrectable coagulopathy because their international normalized ratio stayed above 1.5 even after treatment, who suffered from severe cardiopulmonary instability that made them unable to receive either sedation or general anesthesia, who showed signs of malignancy through pre-procedural imaging or who were pregnant, and who needed immediate emergency biliary drainage for their acute severe cholangitis situation.

### Pre-procedural assessment

The research study conducted thorough pre-procedural evaluations, which included complete clinical assessments that measured symptoms through testing abdominal pain, jaundice, fever, and cholangitis and needed to examine physical health and elderly patients who were more than 60 years old through comprehensive geriatric assessment. The laboratory tests included complete blood count, liver function tests (alanine aminotransferase, aspartate aminotransferase, alkaline phosphatase, gamma-glutamyl transferase, serum albumin, and total and direct bilirubin), the coagulation profile (international normalized ratio), renal function tests (serum creatinine and blood urea), inflammatory markers (erythrocyte sedimentation rate and C-reactive protein (CRP)), and fasting blood glucose. The study used transabdominal ultrasonography as the primary imaging method for all patients, while magnetic resonance cholangiopancreatography (MRCP) was used to create detailed anatomical images, which were needed for certain cases.

### Anesthesia and sedation protocol

The attending anesthesiologist decided which anesthesia method to use after evaluating the patient’s medical condition, the patient’s American Society of Anesthesiologists (ASA) physical status, and the expected difficulty of the surgical procedures. The pre-anesthesia assessment process required medical professionals to conduct airway testing and cardiopulmonary testing and assess existing medical conditions and patient medication use. The anesthesiologist chose deep sedation with propofol-based regimens as the primary sedation method. The medical team used general anesthesia for patients who required it during extended surgery or for patients who had a high risk of aspiration, poor sedation tolerance, or who had multiple health issues. The medical team conducted intra-procedural monitoring through continuous electrocardiography and pulse oximetry, non-invasive blood pressure measurement, and end-tidal carbon dioxide monitoring, which was used whenever it was available. The medical staff used institutional guidelines to implement preventive strategies, which protected patients from developing post-ERCP pancreatitis.

### Interventional procedure

The procedural protocol consisted of five sequential steps. The first step involved conducting standard ERCP with a side-viewing duodenoscope, which included both the TJF-Q180V and JF-260V models (Olympus, Tokyo, Japan) to perform selective CBD cannulation followed by a cholangiogram that determined stone size, number, and location while mapping out ductal anatomy. The second step involved using the SOC operated via the SpyGlass DS system (Boston Scientific). The third step required operators to perform electrohydraulic lithotripsy (EHL) using 1.9-Fr EHL probes (Northgate Technologies), maintaining a 2-mm distance from the stone surface under direct cholangioscopic visualization until the stone was fragmented into pieces smaller than 10 mm that can be removed endoscopically. The fourth step involved using extraction balloon sweepers or Dormia baskets to remove stone fragments. The fifth step involved performing a final occlusion cholangiogram to verify that all ductal pathways had been completely cleared. In all cases, a plastic biliary stent (10 Fr, 7–12 cm) was placed for biliary drainage at the conclusion of the procedure. The endoscopist decided to apply controlled radial expansion (CRE) balloon dilation and sphincteroplasty as additional methods to assist with stone extraction and stricture management [10, 11].

### Outcome measures and definitions

The primary outcome measure was complete stone clearance, which doctors defined through their assessment of final occlusion cholangiogram results after SOC-guided EHL and fragment extraction procedures. Technical success was defined as successful fragmentation of the target stone(s) by EHL under cholangioscopy guidance. Clinical success occurred when doctors confirmed complete ductal clearance through radiological examination. The procedure failed because the team could not achieve complete stone clearance through SOC-guided EHL, which forced them to use different treatment methods. The researchers evaluated safety outcomes by using standardized consensus criteria, including post-ERCP pancreatitis, hemorrhage, perforation, and cholangitis and anesthesia-related adverse events, including hypoxia (SpO_2_ less than 90%), hypotension, and the need for airway intervention [12].

### Statistical analysis

The researchers conducted their statistical analysis using IBM SPSS Statistics for Windows Version 26.0 (IBM Corp. in Armonk, New York, USA). The researchers presented continuous variables as mean values combined with standard deviation (SD) measurements, while they showed categorical variables through frequency counts that included percentage values. The researchers used the chi-square test and Fisher’s exact test to compare categorical variables between groups, while they employed the independent samples Student’s *t-*test for comparing continuous variables. The researchers conducted two-tailed statistical tests, which revealed that results reached statistical significance when P-values dropped below the 0.05 threshold. The researchers conducted a descriptive study because the single-arm study design, together with a small sample size of 25 participants, limited their statistical analysis, which required higher power for subgroup comparisons. To assess the precision of the primary outcome, we performed a post-hoc power calculation. Taking a 70% clearance rate as the benchmark for conventional/alternative extraction techniques in difficult CBD stones, consistent with control-arm outcomes reported in recent randomized trials (63–67%) [5, 13], an exact one-sample binomial test comparing our observed clearance rate (88.0%) against this benchmark yielded a two-sided P-value of 0.051, and the study had approximately 65% power (one-sided α = 0.05) to demonstrate superiority over this benchmark. This reflects the precision limitations inherent to a single-arm cohort of this size; a multicenter cohort of at least 60–70 patients would be required to narrow the 95% confidence interval (CI) to within ± 10 percentage points.

## Results

### Patient demographics and baseline characteristics

The researchers included 25 consecutive patients who suffered from difficult CBD stones in their prospective study, which they conducted from November 2023 until July 2024. Table 1 provides an overview of the demographic and baseline clinical characteristics that define the study population. The average patient age reached 61.8 years with a standard deviation of 6.4 years, and their age ranged from 47 to 75 years. The cohort consisted of 14 female patients who represented 56.0% of the group and 11 male patients who made up 44.0% of the group. Most patients (15 patients, 60.0%) had no significant comorbidities. The study found that hypertension represented the most common comorbidity among patients who had multiple health conditions (seven patients, 28.0%), followed by diabetes mellitus (six patients, 24.0%), hepatitis C virus infection treated with sustained virological response (three patients, 12.0%), ischemic heart disease (one patient, 4.0%), and bronchial asthma (one patient, 4.0%). The most common presenting symptom was right upper quadrant abdominal pain (16 patients, 64.0%), followed by dyspepsia (nine patients, 36.0%), epigastric pain (eight patients, 32.0%), and obstructive jaundice (four patients, 16.0%). Two patients (8.0%) presented with fever consistent with cholangitis. At the index procedure, 16 patients (64.0%) had already undergone cholecystectomy, while nine patients (36.0%) still had their gallbladders present.

**Table 1:** Demographic and Clinical Characteristics of the Study Population (n = 25)

Characteristic	Value (n = 25)
Age (years), mean ± SD	61.8 ± 6.4
Age range (years)	47–75
Sex, n (%)	
Male	11 (44.0)
Female	14 (56.0)
Comorbidities, n (%)	
None	15 (60.0)
Hypertension	7 (28.0)
Diabetes mellitus	6 (24.0)
HCV (treated with SVR)	3 (12.0)
Ischemic heart disease	1 (4.0)
Bronchial asthma	1 (4.0)
Clinical presentation, n (%)	
RUQ abdominal pain	16 (64.0)
Dyspepsia	9 (36.0)
Epigastric pain	8 (32.0)
Jaundice	4 (16.0)
Fever	2 (8.0)
Dark urine	4 (16.0)
Pale stool	4 (16.0)
Nausea/vomiting	3 (12.0)
Gallbladder status, n (%)	
Post-cholecystectomy	16 (64.0)
Gallbladder in situ	9 (36.0)
Previous ERCP, n (%)	
No previous ERCP	1 (4.0)
At least one prior ERCP	24 (96.0)

ERCP: endoscopic retrograde cholangiopancreatography; HCV: hepatitis C virus; RUQ: right upper quadrant; SD: standard deviation; SVR: sustained virological response.

### Pre-procedural laboratory parameters

The complete laboratory tests that were conducted before the procedure are shown in Table 2 for the study population. The average hemoglobin concentration measured 12.68 g/dL with a standard deviation of 1.51 g/dL, and the average total leukocyte count measured 7.26 × 10^3^/µL with a standard deviation of 2.27 × 10^3^/µL. The liver function tests showed results with total bilirubin at 1.15 mg/dL, direct bilirubin at 0.65 mg/dL, alkaline phosphatase at 196.64 U/L, and gamma-glutamyl transferase at 127.40 U/L which showed that most patients experienced biliary obstruction. The average serum albumin level reached 4.21 g/dL with a standard deviation of 0.30 g/dL, which showed that patients maintained sufficient nutritional status. The coagulation parameters achieved normal levels because the mean international normalized ratio reached 1.04 with a standard deviation of 0.09. The renal function assessment showed that renal function remained intact because the average serum creatinine level measured 0.92 mg/dL with a standard deviation of 0.21 mg/dL. The patients showed elevated inflammatory markers because their average erythrocyte sedimentation rate reached 36.92 mm/h with a standard deviation of 8.72 mm/h, and the average CRP level reached 10.00 mg/L with a standard deviation of 14.04 mg/L. The two patients who tested positive for cholangitis showed CRP levels of 48 and 64 mg/L, which showed significant elevation.

**Table 2:** Pre-Procedural Laboratory Parameters of the Study Population (n = 25)

Parameter	Mean ± SD	Reference range	P-value
Hemoglobin (g/dL)	12.68 ± 1.51	12.0–16.0	< 0.001
Total leukocyte count (× 10^3^/µL)	7.26 ± 2.27	4.0–11.0	0.602
Platelet count (× 10^3^/µL)	269.20 ± 97.47	150–400	0.769
ESR (mm/h)	36.92 ± 8.72	0–20	< 0.001
CRP (mg/L)	10.00 ± 14.04	0–6	0.020
INR	1.04 ± 0.09	0.8–1.2	0.036
AST (U/L)	29.92 ± 16.43	10–40	0.147
ALT (U/L)	34.76 ± 19.11	7–56	0.402
Serum albumin (g/dL)	4.21 ± 0.30	3.5–5.0	0.511
Direct bilirubin (mg/dL)	0.65 ± 0.66	0.0–0.4	0.002
Total bilirubin (mg/dL)	1.15 ± 0.75	0.2–1.2	0.006
ALP (U/L)	196.64 ± 92.92	40–129	< 0.001
GGT (U/L)	127.40 ± 59.05	9–48	< 0.001
Serum creatinine (mg/dL)	0.92 ± 0.21	0.6–1.2	0.638
Blood urea (mg/dL)	31.92 ± 10.42	15–45	0.366

ALP: alkaline phosphatase; ALT: alanine aminotransferase; AST: aspartate aminotransferase; CRP: C-reactive protein; ESR: erythrocyte sedimentation rate; GGT: gamma-glutamyl transferase; INR: international normalized ratio; SD: standard deviation.

### Stone characteristics and imaging findings

Table 3 displays the stone characteristics together with imaging results of the study participants. The mean largest stone diameter reached 15.6 mm with a standard deviation of 3.3 mm, and a size range from 5.0 to 22.0 mm. The study found that 11 patients had single stones while 14 patients displayed multiple stones. All patients had stones located inside the CBD. The common hepatic duct was found to have stones in four patients who made up 16.0% of the total. Fifteen patients who made up 60.0% of the study group had stones that exceeded 15 mm in size. A stone-to-duct diameter ratio exceeding 1 was documented in seven patients (28.0%). Impacted stones were present in one patient (4.0%). Complex cases included one patient who had a proximally migrated fully covered self-expanding metal stent with stones and one patient who had multiple biliary strictures with stones. All 25 patients underwent pre-procedural transabdominal ultrasonography, which achieved a 100.0% success rate, while 23 patients underwent MRCP testing, which achieved a 92.0% success rate. Biliary dilation, defined as CBD diameter exceeding 8 mm, was present in 24 patients who made up 96.0% of the total patient group (Figures 1–6).

**Table 3:** Stone Characteristics and Imaging Findings (n = 25)

Characteristic	Value (n = 25)
Largest stone size (mm), mean ± SD	15.6 ± 3.3
Stone size range (mm)	5.0–22.0
Number of stones, n (%)	
Single stone	11 (44.0)
Multiple stones	14 (56.0)
Stone location, n (%)	
Common bile duct	25 (100.0)
Common hepatic duct	4 (16.0)
Intrahepatic ducts	4 (16.0)
Stone size category, n (%)	
> 15 mm	15 (60.0)
10–15 mm	7 (28.0)
< 10 mm	3 (12.0)
Stone/duct ratio > 1, n (%)	7 (28.0)
Impacted stones, n (%)	1 (4.0)
Biliary dilation on imaging, n (%)	24 (96.0)
Pre-procedural imaging, n (%)	
Transabdominal ultrasound	25 (100.0)
MRCP	23 (92.0)

CBD: common bile duct; MRCP: magnetic resonance cholangiopancreatography; SD: standard deviation.

**Figure 1:**
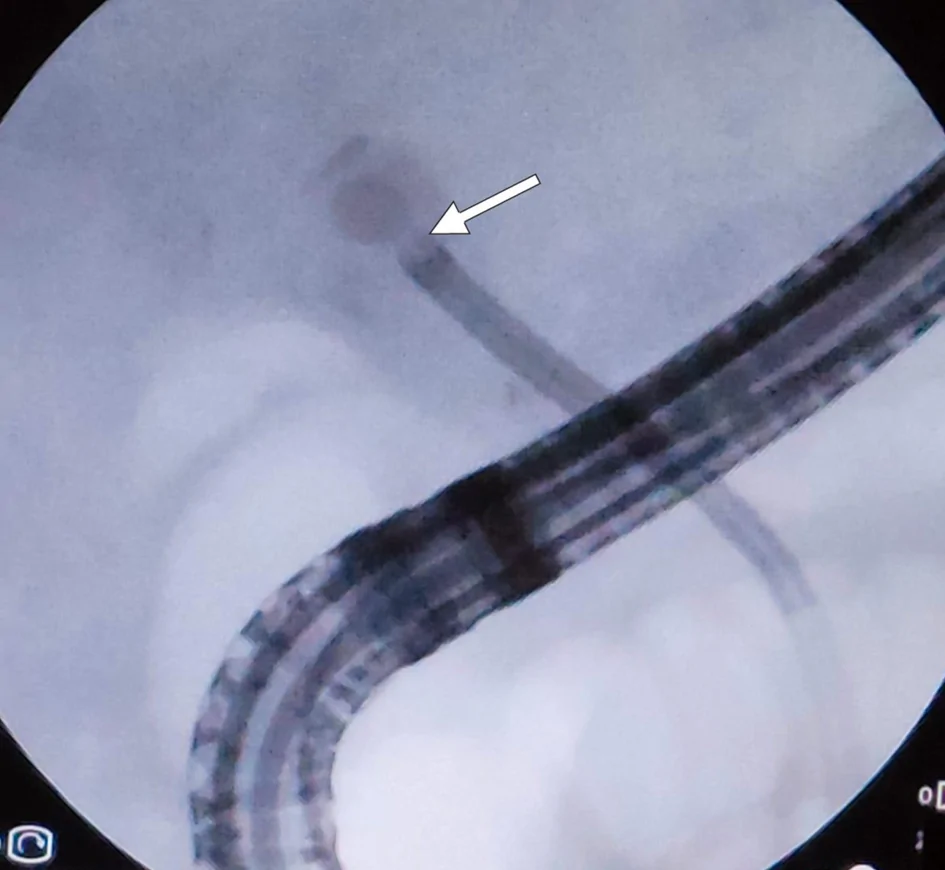
Fluoroscopic image obtained during ERCP demonstrating the single-operator cholangioscope (SpyGlass DS; Boston Scientific, Marlborough, MA, USA) positioned in direct contact with the target CBD stone; the arrow indicates the tip of the cholangioscope abutting the stone surface. CBD: common bile duct; ERCP: endoscopic retrograde cholangiopancreatography.

**Figure 2:**
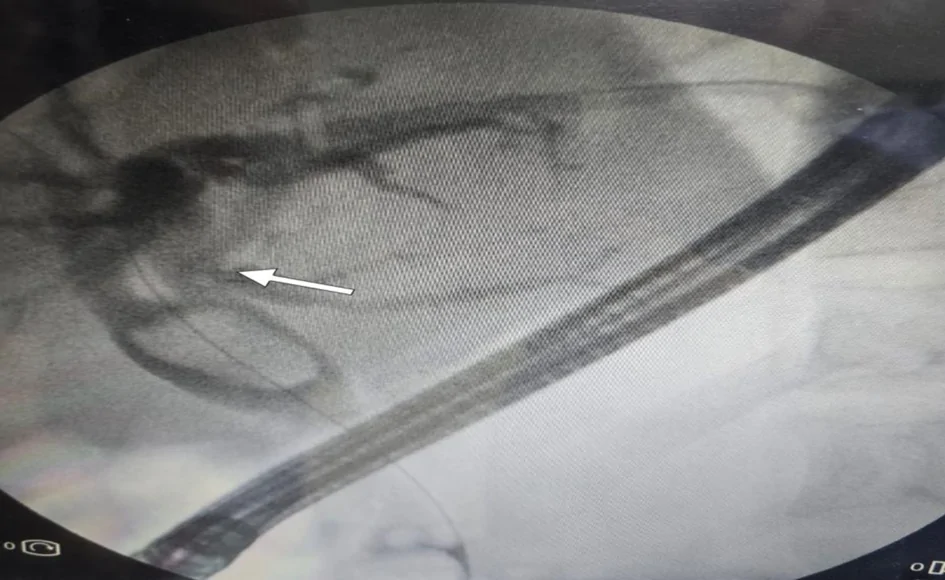
Fluoroscopic cholangiogram showing a large filling defect within the CBD, consistent with a difficult CBD stone; the arrow marks the stone’s proximal margin. CBD: common bile duct.

**Figure 3:**
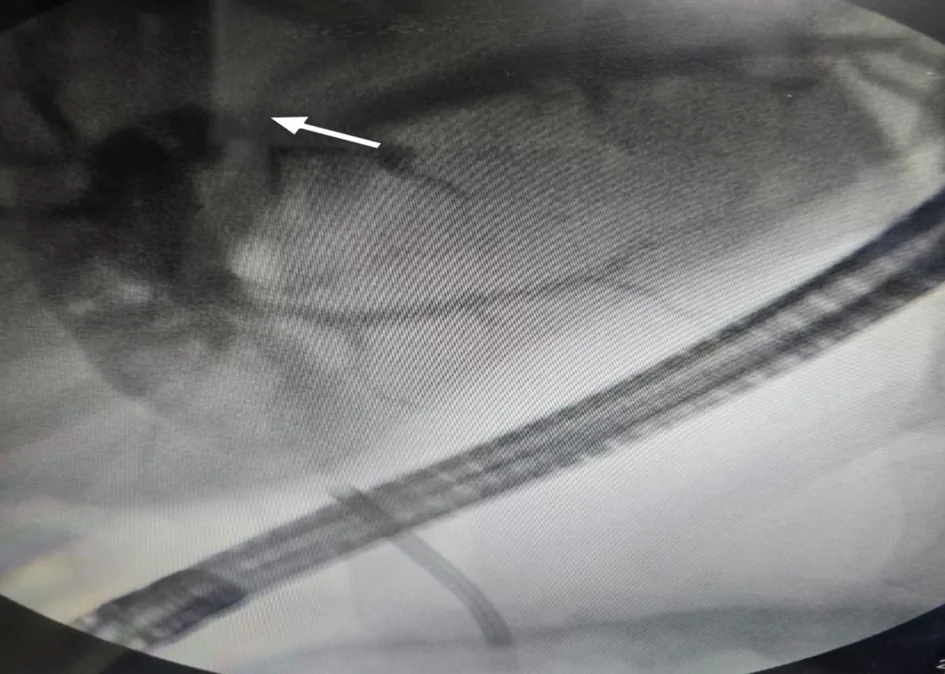
SpyGlass DS single-operator cholangioscope advanced into the CBD to perform EHL. The arrow indicates the cholangioscope tip within the ductal lumen. CBD: common bile duct; EHL: electrohydraulic lithotripsy.

**Figure 4:**
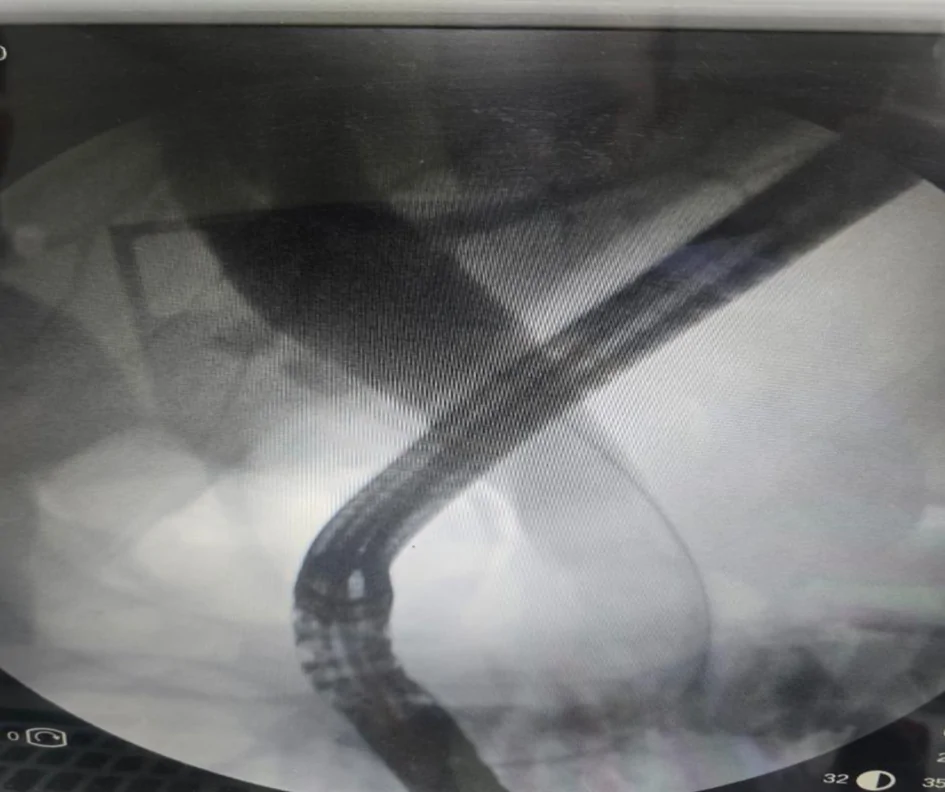
Occlusion cholangiogram, obtained after SOC-guided EHL, confirming complete clearance of the CBD with no residual filling defect. CBD: common bile duct; EHL: electrohydraulic lithotripsy; SOC: single-operator cholangioscopy.

**Figure 5:**
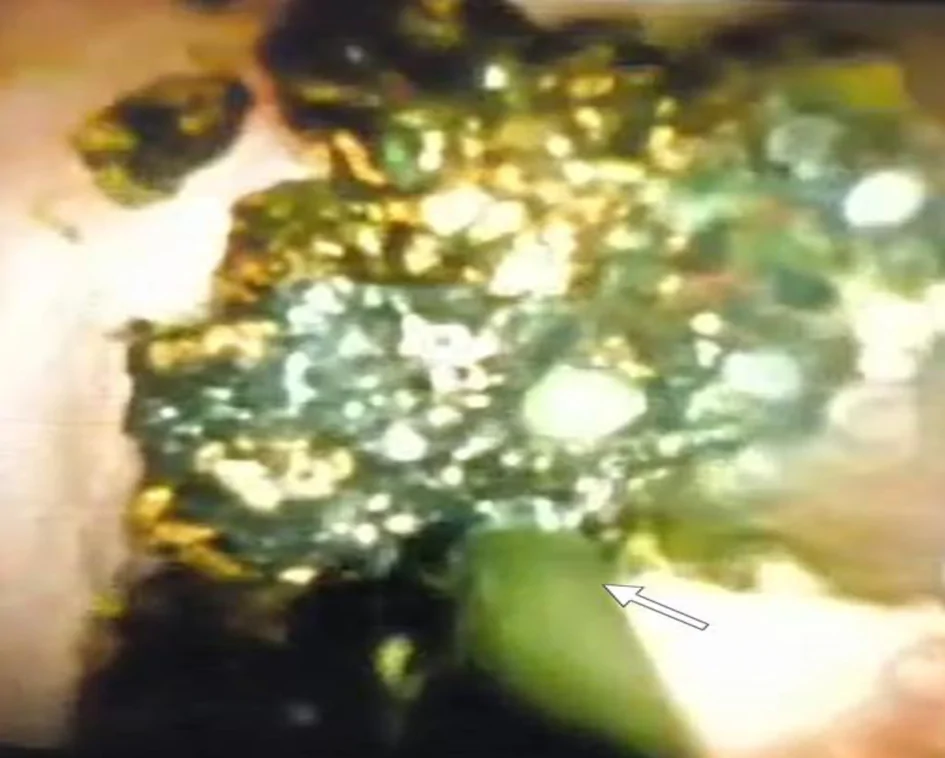
Direct cholangioscopic view during EHL probe lithotripsy, showing the EHL probe (arrow) applied to the stone surface under direct visualization through the single-operator cholangioscope. EHL: electrohydraulic lithotripsy.

**Figure 6:**
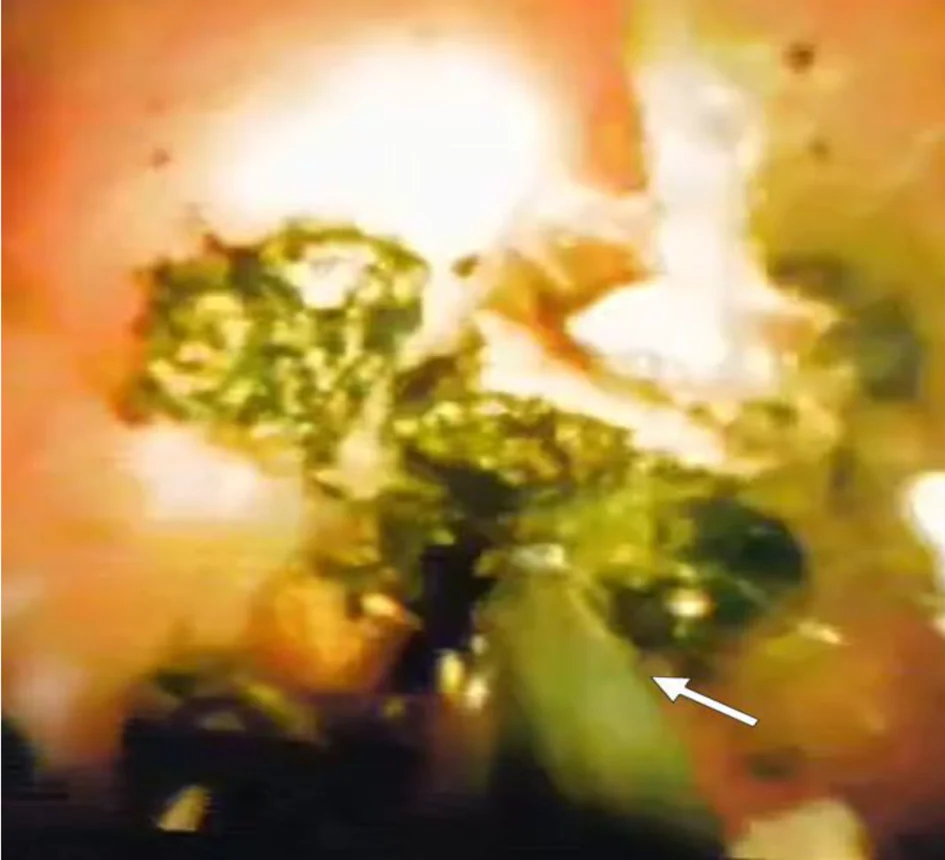
Cholangioscopic image showing fragments of stones in front of EHL probe (arrow), generated by EHL immediately prior to extraction with a balloon sweeper or Dormia basket. EHL: electrohydraulic lithotripsy.

### Procedural outcomes

Table 4 presents a summary of the achieved procedural results together with their associated technical specifications. The clinical success rate reached 88.0% (95% CI: 68.8–97.5%) because 22 out of 25 patients achieved complete stone clearance. The technical success rate reached 88.0% because 22 patients successfully fractured their stones through EHL technology under cholangioscopy inspection. The procedure experienced failure for three patients. The three failed cases showed their complete details through analysis, which showed that the first failed case involved a 75-year-old female patient who had multiple stones situated in the CBD and common hepatic duct and both sides of the hepatic ducts. The second failed case involved a 62-year-old female patient who had a periampullary diverticulum with stones located in the mid CBD and right hepatic duct. The third failed case involved a 57-year-old female patient who had multiple barrel-shaped CBD stones. She achieved partial stone fragmentation and extraction but needed another session to achieve complete stone clearance.

**Table 4:** Procedural Outcomes and Complications (n = 25)

Outcome measure	Value (n = 25)
Primary outcome	
Complete stone clearance, n (%)	22 (88.0)
95% CI	68.8–97.5%
Secondary outcomes	
Technical success (fragmentation), n (%)	22 (88.0)
Clinical success (complete clearance), n (%)	22 (88.0)
Procedural failure, n (%)	3 (12.0)
Extraction method, n (%)	
Balloon extraction	22 (88.0)
Dormia basket	4 (16.0)
Adjunctive procedures, n (%)	
CRE balloon dilation	5 (20.0)
Sphincteroplasty	4 (16.0)
Post-procedural stenting, n (%)	
Single plastic stent	20 (80.0)
Double plastic stent	5 (20.0)
Complications, n (%)	
Post-ERCP pancreatitis	0 (0.0)
Hemorrhage	0 (0.0)
Perforation	0 (0.0)
Cholangitis	0 (0.0)
Anesthesia-related adverse events	0 (0.0)

CI: confidence interval; CRE: controlled radial expansion; ERCP: endoscopic retrograde cholangiopancreatography.

The primary stone retrieval method was balloon extraction, which 22 patients (88.0%) used as their main technique. The Dormia baskets functioned as additional retrieval equipment, which four patients (16.0%) used during their procedures. The doctors performed CRE balloon dilation on five patients (20.0%) to assist with stone removal and to treat their biliary strictures. The surgical team performed sphincteroplasty on four patients (16.0%). The surgical team inserted a plastic biliary stent for biliary drainage at the end of the operation in all 25 patients (100.0%), with 20 patients (80.0%) receiving a single stent and five patients (20.0%) receiving two stents. The patient required the removal of a migrated fully covered self-expanding metal stent, which the medical team extracted using SpyBite Max forceps through the cholangioscopy before they could perform EHL procedures.

### Safety profile

The study found that no significant complications which were linked to surgical procedures occurred in the study group. The study reported that there were no instances of post-ERCP pancreatitis, hemorrhage, perforation, or cholangitis. The study found that there were no anesthesia-related adverse events, which included hypoxia, hypotension, and airway intervention requirements. All patients underwent successful stent placement for biliary drainage at the conclusion of the procedure, and no procedure-related mortality occurred.

## Discussion

The single-arm cohort study shows that SOC-based EHL functions as a safe and effective treatment method, which achieves 88.0% complete ductal clearance success in 25 patients who experienced complex biliary stone disease and failed previous ERCP extraction attempts. The research demonstrates an absence of major procedure-related complications, which supports the advanced endoscopic technique’s safety profile when used in a structured multidisciplinary setting with suitable anesthesia support [14]. The research supports social orientation control through lithotripsy SOC-guided treatment as an effective method to treat patients with challenging biliary stones according to existing research evidence [1, 2].

Our study achieved an overall clinical success rate of 88.0%, which matches and surpasses the success rates from previous studies of SOC-guided lithotripsy used to treat challenging CBD stones. Brewer Gutierrez et al (2018) reported a complete clearance rate of 90.3% in a multicenter study of 219 patients with difficult biliary stones treated with digital SOC [1]. Buxbaum et al (2018) conducted a randomized controlled trial that compared cholangioscopy-guided laser lithotripsy with conventional therapy and found that the cholangioscopy group achieved better complete clearance rates [5]. Laleman et al (2017) reported a technical success rate of 86.5% in a single-center prospective study of 52 patients, with complete clearance achieved in most cases [3]. The SOC-guided EHL method showed effective results in our study because it verified all our findings at a tertiary referral center in Egypt.

The mean largest stone diameter in our cohort was 15.6 ± 3.3 mm, which is consistent with the definition of difficult stones employed in the existing literature. The patient group showed 60.0% of their members with stones that exceeded 15 mm in size and 28.0% with a stone-to-duct ratio that reached over 1. These two conditions represent known risk factors which lead to unsuccessful traditional stone removal. The high proportion of patients with prior cholecystectomy (64.0%) and prior ERCP attempts (96.0%) in our cohort reflects the refractory nature of the stone disease in this patient population and underscores the clinical need for advanced therapeutic modalities such as SOC-guided EHL [15]. The observation that most of our patients (24 of 25) had undergone at least one prior ERCP attempt before being referred for SOC-guided lithotripsy highlights the role of this technique as a rescue therapy for stones that are genuinely difficult to manage with standard approaches [16].

The safety profile observed in our study is particularly noteworthy. The absence of any major procedure-related complications, which include post-ERCP pancreatitis, hemorrhage, perforation, and cholangitis, establishes a better result than the complication rates that exist in medical literature for cholangioscopy-guided lithotripsy. The multicenter studies, which examined larger populations, found complication rates that ranged between 2% and 10%, while post-ERCP pancreatitis emerged as the most common negative outcome [1, 6]. The study achieved a zero-complication rate for multiple reasons. The study procedures received dedicated anesthesia support, which resulted in better patient positioning and reduced aspiration risk and sedation-related complications. The implementation of prophylactic measures through careful cannulation technique and stent placement for biliary drainage and ERCP quality indicators created positive safety outcomes [17, 18]. The study maintained its single experienced operator design, which restricted generalizing results but provided uniform procedural execution and a strict study protocol following. All procedures in this cohort were performed by a single experienced endoscopist at a high-volume tertiary referral center, which standardized technique and likely contributed to the favorable safety profile, but also means our results reflect a best-case, expert-operator scenario rather than real-world performance across operators of varying experience. SOC-guided EHL has a recognized learning curve, and outcomes reported in multicenter or multi-operator series are more heterogeneous than those from single-operator series such as ours [19, 20]. Generalizability of our findings should therefore be limited to centers with comparable procedural volume and dedicated anesthesia support, and future multicenter studies incorporating several operators of differing experience levels, ideally with structured competency assessment, are needed to determine how outcomes vary with operator experience.

The three procedural failures observed in our study merit careful consideration. The first failure occurred in a 75-year-old female with an extensive intrahepatic stone burden involving the CBD, common hepatic duct, and bilateral hepatic ducts, which demonstrated that doctors needed to assess how far the disease inside her body had spread. The second failure involved a patient with a periampullary diverticulum, which is a recognized anatomical challenge that can complicate cannulation and stone extraction, and this patient was ultimately referred for surgical management. The third failure involved a patient with multiple barrel-shaped stones in whom partial fragmentation was achieved, but complete clearance required an additional session, which demonstrates that the shape and material of stones will determine how successful procedures will be. These findings are consistent with those reported by Lenze et al (2018), who identified stone size, location, and associated anatomical factors as predictors of treatment success [11], and by Kamiyama et al (2018), who demonstrated the utility of EHL under both ERCP and peroral transluminal cholangioscopy guidance [14].

The study needs to address its practice of placing plastic biliary stents after every surgical operation. The stent placement procedure, which doctors used to drain bile and conduct future tests, created extra work for doctors because they needed to complete this process during every case that proved total clearance. The literature currently investigates whether doctors should use standard stenting procedures or perform targeted stenting after patients undergo cholangioscopy-based lithotripsy procedures [21, 22]. Yuksel et al (2016) showed that using biliary stenting to treat complex CBD stones produces successful results because the stented stones either broke into smaller pieces or moved out of the body naturally after the ductal system expanded through stent use. The process of stent uses in our procedure created extra safety protections because it helped us identify stone fragments that we failed to see during the cholangiogram test, and it enabled us to conduct planned follow-up tests through stent removal and cholangiographic examination of continuous stone clearance. More specifically, routine post-procedural stenting was adopted as an institutional safety protocol for three reasons: (1) it provides prophylactic biliary drainage in a population with a high baseline burden of ductal obstruction and elevated inflammatory markers; (2) it guards against clinically silent retained fragments that may not be visible on occlusion cholangiography, a recognized limitation of fluoroscopic clearance assessment, by allowing staged re-evaluation at the time of stent removal; and (3) it is consistent with practice patterns discussed in the current European Society of Gastrointestinal Endoscopy (ESGE) guideline on difficult CBD stones, which notes that adjunctive stenting is a reasonable, low-morbidity safety measure in complex or borderline-clearance cases [7]. Because every patient in our cohort received a stent, our reported 88.0% clearance figure should be interpreted as clinical clearance rather than strict intra-procedural single-session clearance, since any fragment identified only at stent removal would not have been captured as a same-session failure; future comparative studies should therefore consider a pre-specified, risk-stratified stenting protocol so that true single-session clearance can be isolated as an outcome.

The observation that adjunctive CRE balloon dilation was required in 20.0% of our patients and sphincteroplasty in 16.0% shows that multimodal treatment methods are essential for effectively handling challenging CBD stone cases. The combination of SOC-guided EHL with adjunctive biliary dilation techniques enables treatment of both existing stone damage and any biliary stricture problems, which will stop stone removal. Xu et al (2017) proved that minor endoscopic sphincterotomy followed by large balloon dilation produces effective results for treating large choledocholithiasis cases [23]. The integration of these complementary techniques as a single procedural system provides the highest chance of complete clearance, which results in fewer needed repeat procedures [24, 25].

### Limitations

This study has multiple limitations which need to be recognized. First, the single-center design and relatively small sample size of 25 patients limit the statistical power of our analyses and the generalizability of our findings. Second, the absence of a control group prevents the researchers from making direct comparisons between SOC-guided EHL and other treatment methods, which include laser lithotripsy, mechanical lithotripsy, and ESWL. Third, the single-operator nature of this study introduces the potential for operator-dependent bias because different operators produce different outcomes based on their procedural experience and techniques. The study could not assess stone clearance durability and stone recurrence rates because it did not include any long-term follow-up data. The routine placement of plastic stents in all patients introduces a potential source of bias because it creates a pathway for biliary drainage, which affects the assessment of the true single-session clearance rate, because some patients might have experienced residual fragments which were not detected until after stent placement. The prospective study design, together with the thorough data collection and the real-world characteristics of our study group at the clinic, shows that SOC-guided EHL can effectively treat difficult CBD stones according to clinical evidence from our research [26, 27].

### Conclusion

The study shows that SOC-based EHL functions as a safe and effective method for treating difficult CBD stones because the procedure achieved complete duct clearance in 88.0% of cases without any major procedure-related complications. The study produced positive results, which took place in a real-world clinical environment that treated patients who had previously undergone unsuccessful conventional ERCP extraction procedures. The study found that SOC-guided EHL needs institutional resources and expertise to achieve safe results because the dedicated anesthesia support and experienced single-operator design led to successful outcomes without complications. The research needs to conduct multicenter randomized controlled trials, which involve bigger groups of participants and monitor them over extended periods, to determine the best patient selection methods and evaluate SOC-guided EHL against alternative lithotripsy treatments while measuring the long-term effectiveness of different treatment methods [28–30].

## Data Availability

The datasets used and analyzed during the current study are available from the corresponding author upon reasonable request.
